# Fluoxetine Attenuated Anxiety-Like Behaviors in Streptozotocin-Induced Diabetic Mice by Mitigating the Inflammation

**DOI:** 10.1155/2019/4315038

**Published:** 2019-07-16

**Authors:** Peng Yuan, Jian Zhang, Liang Li, Zhendi Song

**Affiliations:** ^1^Department of General Practice, Qingdao Municipal Hospital, Qingdao, Shandong, China; ^2^Department of Emergency Medicine, Qingdao Municipal Hospital, Qingdao, Shandong, China; ^3^Department of Breast Surgery, The Second Hospital of Shandong University, Jinan, Shandong, China; ^4^Department of General Surgery, Qingdao Municipal Hospital, Qingdao, Shandong, China

## Abstract

Patients with diabetes mellitus (DM) showed an increased risk of anxiety. High anxiety levels are also shown to increase stress of diabetic patients, which may contribute to poor clinical outcomes. The mechanisms underlying the development of anxiety disorders in diabetic patients remain unknown. As a result, there are no available treatments yet. Here, we tested the hypothesis that glial cells in the hippocampal area of DM mice might be responsible for their anxiety-like behaviors. Furthermore, we postulated that treatment with antidepressant, fluoxetine, could reduce anxiety behaviors and prevent the dysregulation of glial cells (oligodendrocyte and astrocyte) in DM mice. Diabetic mice were administered a single injection of streptozotocin (STZ), followed by treatment with fluoxetine. Mice were then tested on Y maze, open field, dark and light transition, and elevated plus maze tests to measure the status of anxiety and cognition. After completing these behavioral tests, mice were sacrificed and western blot was used to detect the oligodendrocyte and astrocyte maker proteins in hippocampal tissues. Emphasis was directed towards adult oligodendrocyte precursor cells (OPCs) and their marker protein to measure their proliferation and differentiation. We found that fluoxetine could effectively mitigate the level of anxiety and attenuate the cognitive dysfunction in diabetic mice. Meanwhile, fluoxetine inhibited astrocyte activation in mice exposed to STZ, prevented the loss of myelin basic protein (MBP), and affected the function of OPCs in these diabetic mice. The results suggested that the changes of these glial cells in the brains of diabetic mice might be related to the high anxiety levels and cognitive deficit in DM mice. Fluoxetine could ameliorate the high anxiety level and prevent cognitive deficit via inhibiting astrocyte activation and repairing the oligodendrocyte damage.

## 1. Introduction

Patients with diabetes mellitus (DM) exhibit anxiety symptoms more often than people without DM [1]. High anxiety level is not only related to poor clinical outcomes and increased complications among patients with DM, especially among elderly patients, but also greatly impacts the quality of life in DM patients [2]. Anxiety symptoms contain excessive worry, irritability, and fatigue, which contribute to the significant impairment in social functioning [3]. However, no specific treatment strategy for the disorder in DM patients has been established so far [1].

Fluoxetine (FLX) is part of a group of new-generation antidepressants called selective serotonin reuptake inhibitors (SSRIs). FLX has a better safety profile since it was shown to be safe and effective in the elderly population and even during pregnancy [4]. FLX has been widely used for treating depression and anxiety disorders in clinic [5]. Previous studies have implied that FLX could mitigate memory and cognitive deficit in animals [5]. Previous report has also indicated that FLX could exert beneficial effects on memory and cognitive function in patients afflicted with mild cognitive impairment [6].

Recent studies have shown that myelin and oligodendrocyte deficit plays a role in the formation of depression- or anxiety-like behaviors [7, 8]. Intervention compounds targeting myelin or oligodendrocyte could effectively mitigate the depressive- and anxiety-like behaviors [7, 8]. White matter reduction was also found in the central nervous system (CNS) of patients with mood disorders [8]. Meanwhile, patients of chronic demyelinating diseases, such as multiple sclerosis (MS), demonstrated increased risk of mood disorders [9]. These findings implied that myelin deficit might play a role in the development of anxiety- and depression-like behaviors. Diabetes has been shown to be associated with myelin abnormalities such as peripheral neuropathy [10]. Diabetic peripheral neuropathy is a devastating complication in many DM patients at later stages [11]. In the CNS, a neuroimaging study also found that DM patients show alterations in metabolites of both brain gray and white matter [12]. Here, we hypothesized that glial cell abnormality in the CNS of DM mice played an important role in the deficit of memory and the formation of anxiety-like behaviors, but FLX could effectively mitigate these symptoms in DM mice by regulating the function of oligodendrocyte and astrocyte.

## 2. Materials and Methods

### 2.1. Animals and Treatments

Eight-week-old male mice of C57BL/6J were used in the study. The mice were kept in standard environment for laboratory animals. All the mice were given one-week acclimation period before any experimental procedure was carried on. Animal protocols were approved by the Animal Care Committee of Qingdao Municipal Hospital, Shandong, China.

Four groups of mice were used in the present study: control (*n* = 10), control plus FLX (5 mg/kg/day; *n* = 10), STZ (150 mg/kg, *n* = 10), and STZ plus FLX (5 mg/kg/day; *n* = 10). STZ and FLX were purchased from Sigma-Aldrich (MO, USA) and Santa Cruz Biotechnology (Dallas, TX). STZ was prepared in distilled sodium citrate solution with pH at 4.5 and FLX was dissolved in sterile water (2 mg/100 ml water). Single-dose intraperitoneal injection of STZ was administrated to cause DM in a mouse model as previously reported [13]. Water containing FLX was given to mice one week ahead of the STZ injection and lasted till the last day of behavioral test. Behavioral tests were performed 3 weeks later after the STZ injection. Body weight of mice was measured twice per week. We sacrificed the mice and collected the brain samples immediately after the behavioral tests were finished.

### 2.2. Open Field

A square box was used for performing the open field test as previously described with minor modification [14]. Briefly, mice were put in a corner of the open field before the test was initiated. The activity of each mouse in the central and peripheral areas was observed. The total time in the central area and distance travelled during the whole test were analyzed.

### 2.3. Elevated Plus Maze

An elevated plus-shaped maze (EPM) with two open and two closed arms (45 cm × 10 cm) was used for this experiment. The height of the maze to the ground was about 50 cm (elevated). Before starting the test, we put the mice in the central area by facing an open arm. During the test, mice were allowed to probe the elevated maze for 5 min [15]. When four paws of the mouse entered the arms, a valid entry was considered and recorded. The total time of each mouse which stayed in the open arms and the number of each mice entering the total open arms in each test session were recorded for statistical analysis.

### 2.4. Dark and Light Transition Test

Dark and light transition test was carried out as previously described [16]. The device is divided into a light and dark box and there is a shuttle door between these two boxes. The walls of the dark box were painted black with a removable black lid on the top. Mouse was put in the dark box covered with the lid and the shuttle door was closed. After 1 minute, the shuttle door was opened to allow the mouse to freely go into the light box. We counted the first latency of the mouse entering the light box as well as the numbers of transitions between the two boxes. The duration of the test was 5 minutes.

### 2.5. Y Maze

The Y maze spontaneous alternation test is a behavioral test to investigate the willingness of mice to probe novel environments. The apparatus consisted of 3 arms diverging at a 120° from the central point. The procedure was carried out as previously reported with minor change [5]. Each mouse was initially put at the end of one arm by facing all and was allowed to probe the Y maze during an eight-minute test session. The sequences of entering the arms and the number of total entries during the period of 8 minutes were recorded. Percentage of alternation was expressed as the number of sequential triplets containing entries in the three arms during the session as a proportion of the maximum possible alternation (equivalent to the number of total entries minus 2) × 100.

### 2.6. Western Blot

The extracted brain hippocampal samples were run by electrophoresis with SDS-PAGE gel. And then, the protein samples were transferred onto nitrocellulose membranes. We blocked the above membranes with 5% skim milk dissolved in TBST buffer and then incubated the membranes with the primary antibodies against the interested proteins, including antibody to glial fibrillary acidic protein (GFAP) (Millipore Corporation, MA, USA), antibody to platelet-derived growth factor receptor-a (PDGFR*α*) (Santa Cruz Biotechnology), anti-2′,3′-cyclic nucleotide 3′-phosphodiesterase (CNPase) (Millipore Corporation, MA, USA), and myelin basic protein (MBP, Santa Cruz Biotechnology, Santa Cruz, CA, USA) for overnight at 4°C. After being washed 3 times, the above membranes were incubated with their corresponding secondary antibodies for another 2 hours under room temperature. We visualized the bands in the membranes by chemiluminescence reaction (Amersham Biosciences, NJ, USA). GAPDH (Abcam, UK) or *β*-actin (Abcam, UK) antibodies were used to detect the protein expression of these housekeeping genes and considered as the loading controls. Results of each protein were expressed as a ratio of it compared to internal control protein.

### 2.7. Enzyme-Linked Immunosorbent Assay (ELISA)

ELISA kits (eBioscience, Thermo Fisher Scientific) were used for the investigation of IL-6 level in brain tissues according to the manufacturer manuals. Samples were loaded into plate wells in duplicate, and the average of two values was used for final analysis. The final value of IL-6 in statistic was expressed as a ratio of their averages compared to total loading protein.

### 2.8. Statistical Analysis

Values in this study were expressed as the mean ± SEM. The significant differences were defined with one-way ANOVA, followed by Newman-Keuls *post hoc* test. We considered a *p* value of less than 0.05 as statistically significant.

## 3. Results

### 3.1. FLX Improved the Working Memory Performance of DM Mice in a Y Maze Test

**Figure 1:**
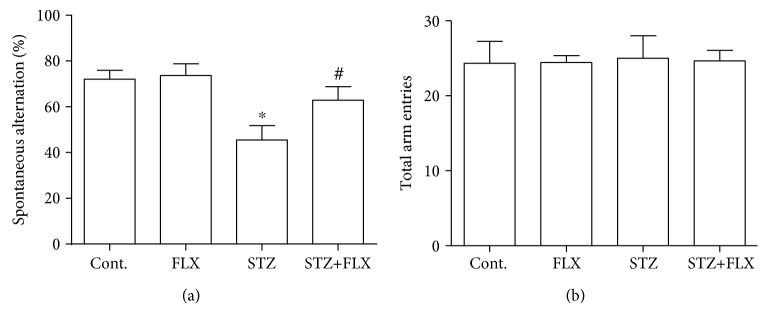
FLX attenuated spatial working memory impairment of DM mice in a Y maze test. (a) STZ induced significant decreased spontaneous alternation of DM mice but FLX could prevent the decrease in DM mice on the working memory performance. (b) The total arm entries among all groups were not influenced by STZ or FLX. All values were expressed as means ± SEM. *n* = 10. ^∗^*p* < 0.01 compared to the control group; ^#^*p* < 0.05 compared to the STZ group.

DM mice reportedly showed significant deficits on memory and cognitive dysfunction [17]. Therefore, we evaluated the FLX influence on the working memory of DM mice by performing a Y maze test. DM mice exerted significant decrease on the spontaneous alternation (Figure 1(a)), which suggested deficits of working memory. However, FLX could effectively prevent the memory loss of DM mice (Figure 1(a)) (*F*_(3, 36)_ = 5.734, STZ group compared to the control group, ^∗^*p* < 0.01; *F*_(3, 36)_ = 5.734, STZ+FLX group compared to the STZ alone group, ^#^*p* < 0.05; one-way ANOVA). Additionally, our results also demonstrated that no significant difference existed on total arm entries between groups (Figure 1(b)), which implicated that the difference on the alternation between groups was not due to the changes of mobility of mice.

### 3.2. FLX Attenuated the Anxiety-Like Behaviors of DM Mice

**Figure 2:**
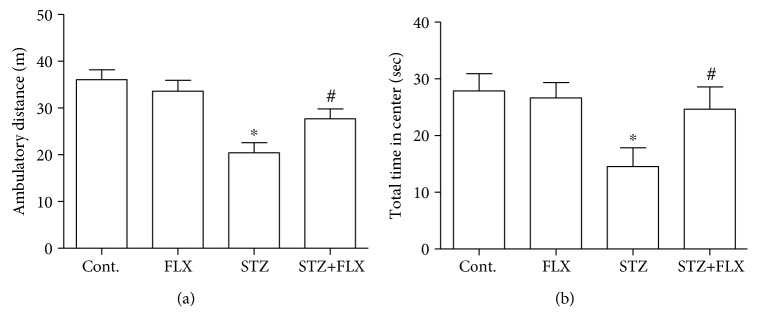
FLX attenuated anxiety-like behaviors of DM mice in the open field. (a) FLX improved the total travel distance of DM mice in the test duration. (b) FLX increased the total time spent of DM mice at the center area. All values were expressed as means ± SEM. *n* = 10. ^∗^*p* < 0.05 or 0.01 compared to the control group; ^#^*p* < 0.05 compared to the STZ group.

**Figure 3:**
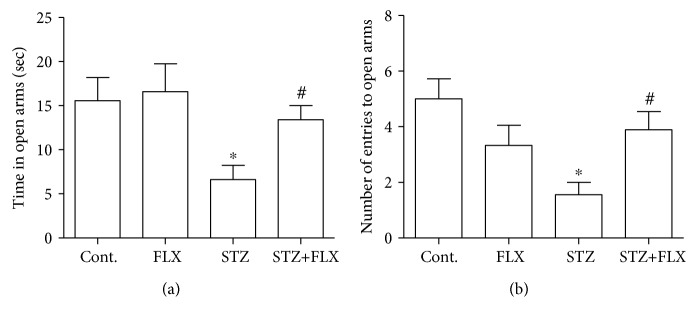
FLX attenuated anxiety-like behaviors of DM mice in an elevated plus maze test. (a) FLX increased the total time spent of DM mice at the open arms. (b) FLX increased the total number of DM mice entering the open arms. All values were expressed as means ± SEM. *n* = 10. ^∗^*p* < 0.05 or 0.01 compared to the control group; ^#^*p* < 0.05 compared to the STZ group.

**Figure 4:**
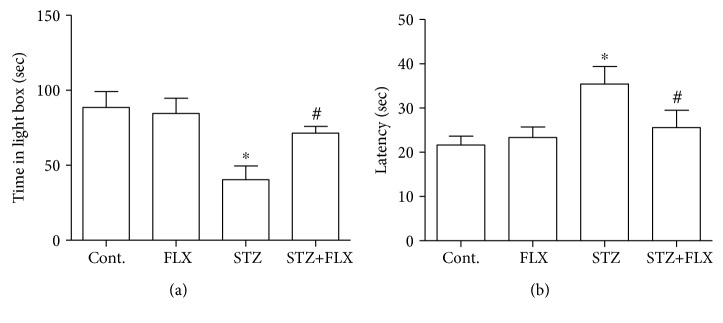
FLX attenuated anxiety-like behaviors of DM mice in the dark and light transition test. (a) FLX increased the total time spent of DM mice at the light box. (b) FLX shortened the latency time of DM mice entering the light box for the first time during the test. All values were expressed as means ± SEM. *n* = 10. ^∗^*p* < 0.05 or 0.01 compared to the control group; ^#^*p* < 0.05 compared to the STZ group.

Next, we measured the anxiety-like behaviors in these DM mice and investigated whether FLX could attenuate these abnormal behaviors. Open field test was employed here to measure the level of anxiety in mice. In this study, the total travel distance and total time spent in the center of each mouse were recorded for the analysis (Figure 2). We found that the DM mice traveled less distance and spent less time in the central area of the open field. Interestingly, mice under FLX treatment showed obvious better performance in distance (*F*_(3, 36)_ = 10.13, STZ group compared to the control group, ^∗^*p* < 0.001; *F*_(3, 36)_ = 10.13, STZ+FLX group compared to the STZ alone group, ^#^*p* < 0.05; one-way ANOVA) and time in the center (*F*_(3, 36)_ = , STZ group compared to the control group, ^∗^*p* < 0.05; *F*_(3, 36)_ = 3.465, STZ+FLX group compared to the STZ alone group, ^#^*p* < 0.05; one-way ANOVA). We also used EPM to test the anxiety level of these DM mice. As shown in Figure 3(a), FLX effectively increased the time spent in the open arms of DM mice in the EPM test, which implied that FLX ameliorated anxiety-like behaviors in the EPM test too (*F*_(3, 36)_ = 3.695, STZ group compared to the control group, ^∗^*p* < 0.05; *F*_(3, 36)_ = 3.695, STZ+FLX group compared to the STZ alone group, ^#^*p* < 0.05; one-way ANOVA). These effects were further validated by the following parameters shown in Figure 3(b) that FLX increased the total numbers of entering the open arms in DM mice (*F*_(3, 36)_ = 4.913, STZ group compared to the control group, ^∗^*p* < 0.01; *F*_(3, 36)_ = 3.695, STZ+FLX group compared to the STZ alone group, ^#^*p* < 0.05; one-way ANOVA). Lastly, light and dark transition test was introduced to further confirm the effects of FLX on the anxiety-like behaviors of these DM mice. Consistent with the results above, FLX prevented the reduction of time spent in the light box of DM mice (Figure 4(a)) (*F*_(3, 36)_ = 5.998, STZ group compared to the control group, ^∗^*p* < 0.01; *F*_(3, 36)_ = 5.998, STZ+FLX group compared to the STZ alone group, ^#^*p* < 0.05; one-way ANOVA). Meanwhile, FLX reduced the latency of DM entering the light box for the first time during the test (Figure 4(b)) (*F*_(3, 36)_ = 3.713, STZ group compared to the control group, ^∗^*p* < 0.05; *F*_(3, 36)_ = 3.713, STZ+FLX group compared to the STZ alone group, ^#^*p* < 0.05; one-way ANOVA). Collectively, all the findings in these above tests implied that FLX could be an effective compound to attenuate the anxiety-like performances and memory loss in DM mice.

### 3.3. FLX Decreased the Levels of GFAP and IL-6 but Increased the MBP, PDGFR*α*, and CNPase Protein Levels of DM Mice

**Figure 5:**
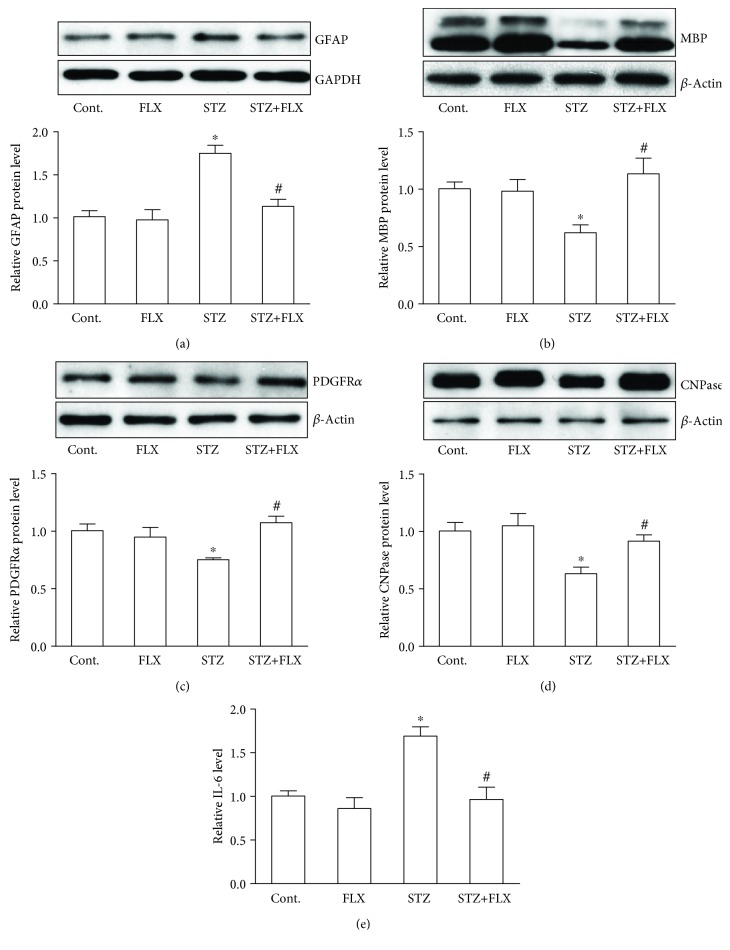
FLX influenced the protein expressions of astrocytes and oligodendrocytes in the CNS of DM mice. (a) FLX reduced the expression level of GFAP. (b) FLX increased the expression level of MBP. (c) FLX increased the expression level of PDGFR*α*. (d) FLX increased the expression level of CNPase. (e) FLX reduced the expression level of IL-6. All values were expressed as means ± SEM. *n* = 5. ^∗^*p* < 0.05 compared to the control group; ^#^*p* < 0.05 compared to the STZ group.

Next, we explored the possible underlying mechanism by which FLX protected DM mice against memory loss and anxiety-like behavior formation. To test whether astrocyte activation was involved in the regulation of FLX on mice exposed to STZ, western blot assays were firstly used with antibody of GFAP, which is a common protein maker of astrocyte activation. As shown in Figure 5(a), the GFAP expression level was increased in STZ-induced DM mice, which could be prevented by the cotreatment of FLX. Meanwhile, we found that the level of IL-6 was significantly upregulated in the brain samples of STZ mice but could be inhibited by FLX treatment (Figure 5(e)) (*F*_(3, 22)_ = 10.31, STZ group compared to the control group, ^∗^*p* < 0.05; *F*_(3, 22)_ = 10.31, STZ+FLX group compared to the STZ alone group, ^#^*p* < 0.05; one-way ANOVA). These two results suggested that FLX could ameliorate the activation of astrocyte in the brain exposed to STZ. Secondly, we investigated whether STZ caused myelin damage in the hippocampal area of the DM mice by detecting the MBP expression level. We found that there was notable reduced expression level of MBP in DM mice but FLX could ameliorate the MBP loss (Figure 5(b)). Thirdly, we asked whether FLX affected the adult OPCs, which usually surround the lesion area of myelin and play important roles during the myelin repair in many pathological scenarios [8, 18]. We focused on two proteins, CNPase and PDGFR*α*. CNPase is an important protein during the development of oligodendrocytes to become myelin-forming cells [19]. PDGFR*α* is one of the feature protein markers of OPCs [20]. As shown in Figures 5(c) and 5(d), STZ treatment led to significant reduction of PDGFR*α* (*F*_(3, 22)_ = 3.31, STZ group compared to the control group, ^∗^*p* < 0.05; one-way ANOVA) and CNPase (*F*_(3, 36)_ = 4.094, STZ group compared to the control group, ^∗^*p* < 0.05; one-way ANOVA) expression but FLX prevented the protein loss of both these proteins, respectively (*F*_(3, 22)_ = 3.31, STZ+FLX group compared to the STZ alone group, ^#^*p* < 0.05; *F*_(3, 36)_ = 4.094, STZ+FLX group compared to the STZ alone group, ^#^*p* < 0.05). Our results indicated that FLX might improve the behavioral performance in DM mice by inhibiting the glial cell activation and protect myelin and OPC loss in the hippocampal area of DM mice.

## 4. Discussion

Recent findings support that the brain is one of the common targets of diabetic complications [21]. The possibility of mood disorders such as depression in patients with DM is doubled than that in patients without DM [22]. Moreover, patients with DM cooccurring with anxiety are at an increased risk of mortality [23]. Anxiety is closely relevant with the dysregulation of the hypothalamic-pituitary-adrenal axis (HPA), which can trigger insulin resistance [24, 25]. Therefore, anxiety or other mood disorders may be a contributing factor for DM through biological and behavioral manners. So far, there are no specific compounds available clinically to treat anxiety or depression in DM patients. Here, we used a STZ-induced DM mouse model to investigate whether FLX could effectively prevent the formation of anxiety-like behaviors and memory deficit in DM mice. We found that DM mice showed obvious anxiety performances in a series of behavioral tests. The results here are consistent with reports from other groups [26, 27]. More importantly, our results also disclosed that FLX treatment could mitigate these abnormal behavioral performances. In the Y maze test, STZ induced significant decreased spontaneous alternation but could be reversed by FLX treatment (Figure 1). These results suggested that FLX not only could protect DM patients against the formation of anxiety-like behaviors but also exert protective effects on memory and cognitive deficit.

Astrocyte activation is reportedly found in the brain of DM mice [27]. Here, we found that astrocyte activation maker protein, GFAP, was notably upregulated on the protein level (Figure 5(a)), which suggested a neuroinflammatory response in the CNS of DM mice. Interestingly, the increase of GFAP was inhibited by FLX cotreatment. At the same time, the level of IL-6 in the mouse brain was increased but could be inhibited by the FLX treatment. These results were consistent with a previous report that FLX could inhibit the activation of astrocytes in animal models of Alzheimer's disease [28]. And then, we explored the beneficial effects of FLX on oligodendrocyte by detecting the myelin protein, MBP. We found that there was an improvement of MBP protein expression accompanying the inhibition of GFAP (Figure 5(b)). These results reminded us to look at another glial cell, oligodendrocyte. Adult OPC proliferation and differentiation are among the key features of myelin regeneration [29]. Two important marker proteins of OPCs were investigated with western blot in the studies that followed. We found that STZ caused reduced expression of PDGFR*α* and CNPase, which could be reversed by FLX (Figures 5(c) and 5(d)). These results suggested that FLX could also regulate the adult OPCs in the hippocampal area of DM mice and improve their behavioral performance.

Neurobehavioral complication in DM patients is a rising problem that needs research attention since the damage to the neurosystem has a significant impact on the medical cost and life quality of DM patients. According to the knowledge we have known so far, there are fewer efforts made to the preclinical setting about this issue. Our study provided evidences that anxiety and memory loss are the essential part of neuropathological changes of DM mice. And the results were in line with a previous report that anxiety-like behaviors could develop in mice with STZ exposure [30]. FLX may be a good candidate to handle these disorders in DM patients by attenuating the astrocyte activation and rescuing the myelin deficit in the CNS. Meanwhile, the beneficial effects could also result from the direct anti-inflammatory role since FLX already was shown to be able to inhibit the inflammatory response from astrocytes [28]. Further studies involving the other inflammatory cells in the CNS, like microglia, are needed to elaborate the details of cellular response in STZ-induced DM mice.

## 5. Conclusions

The principal finding of this study is that FLX was able to exert its preventive effects on memory loss and anxiety behaviors in DM mice. The beneficial effects of FLX may be due to its regulation on astrocyte activation and oligodendrocyte dysfunction in the CNS of DM mice. Whether FLX can provide benefit to patients suffering from DM warrants further investigation in clinical setting.

## Data Availability

The data used to support the findings of this study are available from the corresponding authors upon request.
